# Adaptation of African swine fever virus to porcine kidney cells stably expressing CD163 and Siglec1

**DOI:** 10.3389/fimmu.2022.1015224

**Published:** 2022-10-27

**Authors:** Qi Gao, Yunlong Yang, Yizhuo Luo, Jiachen Zheng, Lang Gong, Heng Wang, Yongzhi Feng, Ting Gong, Dongdong Wu, Ruixia Wu, Xiaoyu Zheng, Zezhong Zheng, Guihong Zhang

**Affiliations:** ^1^ Guangdong Provincial Key Laboratory of Zoonosis Prevention and Control, College of Veterinary Medicine, South China Agricultural University, Guangzhou, China; ^2^ African Swine Fever Regional Laboratory of China (Guangzhou), Guangzhou, China; ^3^ Maoming Branch, Guangdong Laboratory for Lingnan Modern Agriculture, Maoming, China; ^4^ Research Center for African Swine Fever Prevention and Control, South China Agricultural University, Guangzhou, China; ^5^ Key Laboratory of Animal Vaccine Development, Ministry of Agriculture and Rural Affairs, Guangzhou, China

**Keywords:** African swine fever virus, whole gene transcription, adaptation, CD163, Siglec1

## Abstract

African swine fever virus (ASFV) is a complex large DNA enveloped virus that causes African swine fever (ASF) with a fatality rate of up to 100%, seriously threatening the global swine industry. Due to the strict cell tropism of ASFV, there is no effective *in vitro* cell line, which hinders its prevention and control. Herein, we analyzed genome-wide transcriptional profiles of ASFV-susceptible porcine alveolar macrophages (PAMs) and non-susceptible cell lines PK15 and 3D4-21, an found that PAM surface pattern recognition receptors (PRRs) were significantly higher and common differential genes were significantly enriched in phagocytosis compared with that observed in PK15 and 3D4-21 cell lines. Therefore, endocytosis functions of host cell surface PRRs may play key roles in ASFV infection *in vitro*. ASFV was found to be infective to PK15 and 3D4-21 cell lines overexpressing CD163 and Siglec1, and to the PK15^S1-CD163^ cell line stably expressing CD163 and Siglec1. However, the PK15 and 3D4-21 cell lines overexpressing CD163 or Siglec1 alone were not infectious. Simultaneous interference of CD163 and Siglec1 in PAMs with small interfering RNA (siRNA) significantly reduced the infectivity of ASFV. However, siRNA interference of CD163 and Siglec1 respectively did not affect ASFV infectivity. ASFV significantly inhibited IFN expression levels in PAMs and PK15^S1-CD163^ cells, but had no effect on PK15 and 3D4-21 cell lines. These results indicate that CD163 and Siglec1 are key receptors for ASFV-infected host cells, and both play a synergistic role in the process of ASFV infection. ASFV inhibits IFN expression in susceptible cells, thereby downregulating the host immune response and evading the immune mechanism. The discovery of the ASFV receptor provides novel ideas to study ASFV and host cell interactions, pathogenic mechanisms, development of receptor blockers, vaccine design, and disease resistance breeding.

## Introduction

African swine fever (ASF) is an acute, lethal, highly contagious infectious disease of pigs caused by the African swine fever virus (ASFV), with a mortality rate of up to 100% (1, 2). ASF was first reported in Kenya, East Africa in 1921. Until August 2018, when genotype II ASFV was first introduced to China, causing huge economic losses to the pig industry, there was no effective vaccine for ASF prevention and control (3, 4). ASFV belongs to the Asfivirus genus of the Asfivirus family. It is a linear double-stranded DNA virus with a genome of 170–193 kb (5). It encodes more than 150 proteins that play important roles in virus assembly, replication, and immune evasion (6, 7). There is no effective *in vitro* subculture cell line for ASFV. Porcine monocyte-macrophages are the main target cells of ASFV infection, and it is also the only *in vitro* system that can simulate natural ASFV infection. All ASFV isolates are easy to grow in this system (8). However, since it is a primary cell, the production procedure is complex and costs are high. Furthermore, the pig’s internal environment is complex, with large individual differences, and maternal antibodies in weaned piglets may interfere with the replication of ASFV. Therefore, these issues limit the development of ASFV mechanistic research. In contrast, the *in vitro* cell infection model does not have above-mentioned limitations and is more suitable to study interaction mechanisms between ASFV and host cells. Moreover, the establishment of a stable and efficient *in vitro* cell culture model of ASFV is also helpful for the development of effective vaccines. Several passaged cell lines supporting ASFV replication have been reported, such as A4C2/9K, WSL, MA104, ZMAC-4, IPKM, Vero, MS, CV-1, 3D4-21, and COS-1 (9–13). These cell lines all meet the scientific purpose of serial passage of ASFV to a certain extent, but it remains to be determined whether gene expression is lost or immunogenicity changes after serial passage of the virus.

Due to the large size of ASFV virions, it is difficult to enter cells. Therefore, it is easier to enter macrophages *via* phagocytosis. ASFV mainly enters host cells through receptor-mediated endocytosis, classical clathrin-mediated endocytosis, and actin-mediated endocytosis (14). Viral receptors are molecular complexes that are located on the surface of host cells and can be recognized and bound by virus-specific proteins, thereby causing viral infection (15). Receptors play an extremely important role in the process of cellular information transmission. Binding of viruses to receptors is the first step of virus infection. ASFV can infect susceptible cells by binding to cellular receptors. CD163 is a 130 kDa type-I transmembrane glycoprotein (16–18), its extracellular domain is composed of nine scavenger receptor cysteine-rich (SRCR) domains of about 100–110 amino acid residues (19). Current studies have shown that CD163 is considered one of the most likely receptors for ASFV. The expression of CD163 increases when porcine monocytes (PBMCs) differentiate into mature macrophages, and ASFV infection also increases. More ASFV-infected cells were found in PBMCs expressing CD163 than ASFV-infected cells lacking this receptor (20, 21). Moreover, when macrophages were incubated with anti-CD163-specific antibodies, ASFV binding to cells was reduced by more than 50%. However, the role of CD163 in ASFV infection is controversial, and studies have shown that stable expression of CD163 in non-susceptible cell lines is not sufficient to increase susceptibility to ASFV (22). CD163 knockout pigs are resistant to the Georgia07 strain of ASFV, but can still be infected (23). Siglec1 or Sialodhesin (Sn), also known as CD169, is a sialic acid-binding immunoglobulin-like lectin receptor, mainly expressed in macrophages of different tissues, but not monocytes (24). Siglec1 is produced by IFN-α-mediated membrane glycoprotein and contains 17 Ig-like domains extracellularly and the sialic acid-binding domain located within the distal membrane domain (25). Siglec1 is reportedly a regulator of inflammation and immune responses and can phagocytose cells by interacting with other receptors (26). Siglec1 has been described as a receptor for various viruses, such as in macrophage-mediated endocytosis of porcine reproductive and respiratory syndrome virus (PRRSV) (27). Based on the characteristics of ASFV with monocyte-macrophage tropism, we performed bioinformatics analysis by comparing the genome-wide transcriptional profiles of its susceptible porcine alveolar macrophages (PAMs) and non-susceptible cell lines PK-15 and 3D4-21. We screened and verified the function of differential genes. Combined with the biological function test of the *in vitro* culture system of the PK-15 S1-CD163 cell line, potential receptors of ASFV infection and the main determinants of proliferation were initially revealed *in vitro*, which laid a theoretical foundation for the establishment of a perfect ASFV *in vitro* cell infection and proliferation system. We believe that this research will be useful for rapid treatment and diagnosis of ASFV, as well as providing a platform for research into developing an *in vitro* model to further our understanding regarding ASFV and the underlying mechanisms of infection.

## Materials and methods

### Cells and viruses

Porcine kidney 15 (PK15) cells and PK15^S1-CD163^ were grown in Dulbecco’s modified Eagle’s medium (DMEM, Gibco, Waltham, MA, USA) supplemented with 10% fetal bovine serum (FBS, Gibco, Waltham, MA, USA) at 37°C with 5% CO_2_. The PK15^S1-CD163^ cell line was a gift from Prof. Hans J. Nauwynck, Department of Virology, Immunology and Parasitology, Faculty of Veterinary Medicine, Ghent University, Belgium. The cell line is porcine kidney cells stably expressing CD163 and Siglec1. Primary porcine alveolar macrophages (PAMs) were collected from 20-30-day-old specific pathogen free pigs. We confirm that the ethical policies of the journal, as noted on the journal’s author guidelines page, have been adhered to and the appropriate ethical review committee approval has been received. The US National Research Council’s guidelines for the Care and Use of Laboratory Animals were followed. PAM 3D4/21 cells were maintained in RPMI 1640 medium with l-glutamine (Gibco, Waltham, MA, USA) supplemented with antibiotics (100 U/mL of penicillin and 100 mg/mL streptomycin, Gibco, Waltham, MA, USA) and 5% FBS at 37°C with 5% CO_2_. The high virulence, hemadsorbing ASFV isolate GZ201801 (GenBank: MT496893.1) was isolated in Guangzhou, China, was p72 genotype II, and was preserved in the Infectious Diseases Laboratory of South China Agricultural University.

### Reagents and antibodies

Primers and probes for amplifying genes were synthesized by Invitrogen (Invitrogen, Waltham, MA, USA), and reagents were purchased for downstream experiments, including Lipofectamine 2000 (Invitrogen, Waltham, MA, USA), AceQ^@^ Universal U+ Probe Master Mix V2 (Vazyme, Nanjing, China), and ChamQ Universal SYBR qPCR Master Mix (Vazyme, Nanjing, China). The murine monoclonal p30 antibody was prepared by our laboratory and used in both western blotting and immunofluorescence assays (IFAs). Several antibodies and stains were purchased, including α-tubulin rabbit polyclonal antibody (Beyotime, Shanghai, China), DAPI (Beyotime, Shanghai, China), anti-CD136 rabbit monoclonal (Abcam, Cambridge, UK), anti- sialoadhesin/CD169 mouse monoclonal (Abcam, Cambridge, UK), and goat anti-rabbit IgG Alexa Fluor^®^ 488 (Abcam, Cambridge, UK). siRNA was synthesized by Guangzhou Ribo (CD163 siRNA: TAGTTCTCTTGGAGGAAAAGACA, Siglec1 siRNA: AAGCTCAAAGACCAGAAACGTGT). We purchased AxyPrep™ Body Fluid viral DNA/RNA Miniprep Kit from AXYGEN (AXYGEN, Hangzhou, China). Porcine IFN-β ELISA kit (Solarbio, Beijing, China).

### Virus infection

PAMs, PK15, 3D4-21 and PK15^S1-CD163^ cells in T25 cell culture flasks were infected with 1 MOI ASFV and incubated for five days at 37°C with 5% CO_2_, respectively. Cells and supernatant were harvested and stored at -80°C. For subsequent passaging, 1 mL of the previously passaged virus was used to infect cells in T25 cell culture flasks. Finally, five passages were successively performed under the same conditions. Following incubation for 2 h at 37°C with 5% CO_2_, the culture medium was discarded, cells were washed twice with phosphate-buffered saline (PBS), and incubated at 37°C with 5% CO_2_. The cell-culture supernatants or cells were collected at 12, 24, 36, 48, and 72 h post-infection (hpi), stored at -80°C and analyzed to detect extracellular and intracellular virions. Viral genome copy numbers were analyzed using Quantitative real-time polymerase chain reaction (RT-qPCR).

### Real-time qPCR analysis

ASFV genomic DNA was extracted from cell supernatants or cells using an AxyPrep™ Body Fluid viral DNA/RNA Kit. A total of 2 µL of DNA was used for real-time PCR assay using AceQ Universal U+ Probe Master Mix V2 (Vazyme, Nanjing, China). The relative quantity of viral DNA was determined using the CADC p72 primers and a probe experiment. Total RNA was isolated by RNAiso Plus (Takara, 9108). RNA was reverse transcribed into cDNA using the HiScript II 1st Strand cDNA Synthesis Kit (+gDNA wiper) (Vazyme, China, R212-02). A total of 1 μL of cDNA was used for real-time PCR assay using ChamQ Universal SYBR qPCR Master Mix (Vazyme, China, Q711-02). The relative quantity of cell RNA was determined by performing a comparative Ct (ΔΔCt) experiment using GAPDH as an endogenous control. qPCR assays were performed on a Bio-Rad CFX96 real-time PCR machine (Bio-Rad, Hercules, CA, USA) according to the manufacturer’s instructions. Gene-specific primer and probe sequences are listed in **Supplementary Table 1**.

### RNA isolation, cDNA library preparation, and sequencing

PAMs, PK15, and 3D4-21 cells were harvested from T75. The total RNA was extracted from the cells using RNAiso Plus (TAKARA, Kyoto, Japan), according to the manufacturer’s instructions. RNA quantity and purity were assessed using a Thermo NanoDrop Lite spectrophotometer (Thermo Fisher Scientific, MA, USA). Samples were sent to Novogene (Beijing, China) for the removal of ribosomal RNA to construct strand-specific libraries. First, ribosomal RNA was removed from total RNA, and RNA was fragmented into 250**–**300 bp sections. The fragmented RNA was used as a template and a random oligonucleotide was used as a primer to synthesize the first strand of cDNA. Thereafter, RNase H was used to synthesize the first strand of cDNA. The RNA strand was degraded, and the second strand of cDNA was synthesized using dNTPs (dUTP, dATP, dGTP and dCTP) as raw materials under the DNA polymerase I system. The purified double-stranded cDNA was end-repaired, A-tailed, and connected to sequencing adapters, and AMPure XP beads were used to screen cDNAs of approximately 200 bp. The U-containing cDNA second strand was then degraded using USER enzyme, and finally PCR amplification was performed to obtain a library.

### Data analysis of RNA-Seq

The standard analysis process of RNA-seq mainly includes quality control, alignment, splicing, screening, quantification, difference significance analysis, and functional enrichment. The core of RNA-seq analysis is the significance of gene expression differences. Statistical methods are used to compare gene expression differences under two or more conditions, to identify differential genes associated with certain conditions, and then to further analyze the biology and significance of these differentially expressed genes (DEGs). Unigenes with fold change > 2 and Q value ≤ 0.05 were considered significantly differentially expressed. Using the Novogene analysis system, clustering heat map, Venn, gene ontology (GO), and Kyoto Encyclopedia of Genes and Genome (KEGG) analysis, analysis of DEGs and proteins were performed. Functional annotation and pathway analysis of DEGs were performed using the GO and KEGG databases and the resulting graphs were presented.

### Plasmid and siRNA transfection

Firstly, 10 μmol siRNA and 1.5 μL lipo2000 were diluted with 50 μL optim, respectively. These were incubated at 25°C for 5 min. After incubation, the siRNA diluent was mixed with the lipo2000 diluent (total volume was about 100 μL), gently mixed, and incubated at 25°C for 20 min. The mixed cultures were added to the complete medium of the 24-well plate. The plates were incubated at 37°C with 5% CO_2_ for 24 h to perform subsequent virus infection experiments and test the inhibitory effect.

### Immunofluorescence assay

Cells infected with ASFV at a multiplicity of infection (MOI) of 1 were seeded on a 24-well plate and incubated with ASFV p30 protein monoclonal antibody, which was previously diluted with 2% bovine serum albumin (BSA) at a ratio of 1:500. At 24 h post-infection, the cells were washed five times with PBS (1 mL each time), fixed in 500 μL 3.7% paraformaldehyde for 30 min at 25°C, permeabilized in 1 mL 0.1% (w/v) Triton X-100 for 20 min at 25°C, and then incubated in the dark with a secondary antibody diluted with 2% BSA (1:200) for 1 h at 37°C in a humid chamber. Thereafter, cell nuclei were stained with 4′,6-diamino-2-phenylindole (DAPI) at 25°C for 5 min and washed thrice with PBS. Cell fluorescence was observed using an immunofluorescence microscope (Nikon, Tokyo, Japan).

### Western blot analysis

For western blot analysis, cells were lysed in RIPA buffer (Beyotime) and denatured by adding 4× Laemmli sodium dodecyl sulphate-polyacrylamide gel electrophoresis (SDS-PAGE) buffer (containing DL-dithiothreitol), followed by heating for 15 min at 100°C. The proteins were then separated on SDS-PAGE gels and transferred onto nitrocellulose membranes using a Trans-Blot Turbo rapid transfer system (Bio-Rad), according to the manufacturer’s instructions. The membranes were blocked in 5% defatted milk (dissolved in Tris-buffered saline (TBS)) for 1 h at 37°C and then incubated with a primary antibody for 1 h at 25°C or 12 h at 4°C. The membranes were then washed thrice (5 min per wash) using a wash buffer (TBS containing 0.1% Tween-20) and incubated with an IRDye^®^ 800CW secondary antibody for 1 h at 37°C. The membranes were washed thrice in wash buffer and imaged using an Odyssey Imaging System (LI-COR, USA) to visualize the protein bands. α-Tubulin was used as a loading control.

### Flow cytometry

Flow cytometry analysis of PAMs treated with CD163 siRNA (100 nM) and Siglec1 siRNA (100 nM) for 24 h to label sialoadhesin/CD169 and CD163 was conducted. Goat anti rabbit IgG (Alexa Fluor^®^ 488) was used as the secondary antibody. Cells were fixed with 4% paraformaldehyde and permeabilized with 0.1% Tween-20.

### ELISA

Cell supernatants were collected and assayed for porcine IFN-β using a porcine IFN-β ELISA kit. The measured value was compared with the standard according to the manufacturer’s instructions.

### Statistical analysis

Pathway analysis and functional annotation of DEGs and differentially expressed proteins identified by transcriptomics and proteomics were performed using the KEGG, GO, and KOG databases, respectively. The STRING method was used for protein network interaction analysis. The SPSS software package (SPSS for Windows version 13.0, SPSS Inc., Chicago, IL, USA) was used to perform statistical analysis of data obtained during the experiment. The difference between the experimental group and the control group was analyzed using one-way ANOVA using GraphPad Prism 8 (GraphPad Software, San Diego, CA, USA). Values are expressed in bar graphs as the mean ± standard deviation (SD) of at least three independent experiments. Statistical significance was set at * p < 0.05, *** p < 0.01, and **** p < 0.001.

### Biosafety statement and facility

All experiments involving live ASFV were conducted within the biosafety level 3 (BSL-3) facility at South China Agricultural University. The viruses were inactivated in a BSL-3 laboratory, and the inactivated samples were transferred to a BSL-2 laboratory for the extraction and detection of ASFV genomic DNA\RNA.

## Results

### Biological properties of ASFV in PAM, PK15, and 3D4-21 cells

**Figure 1:**
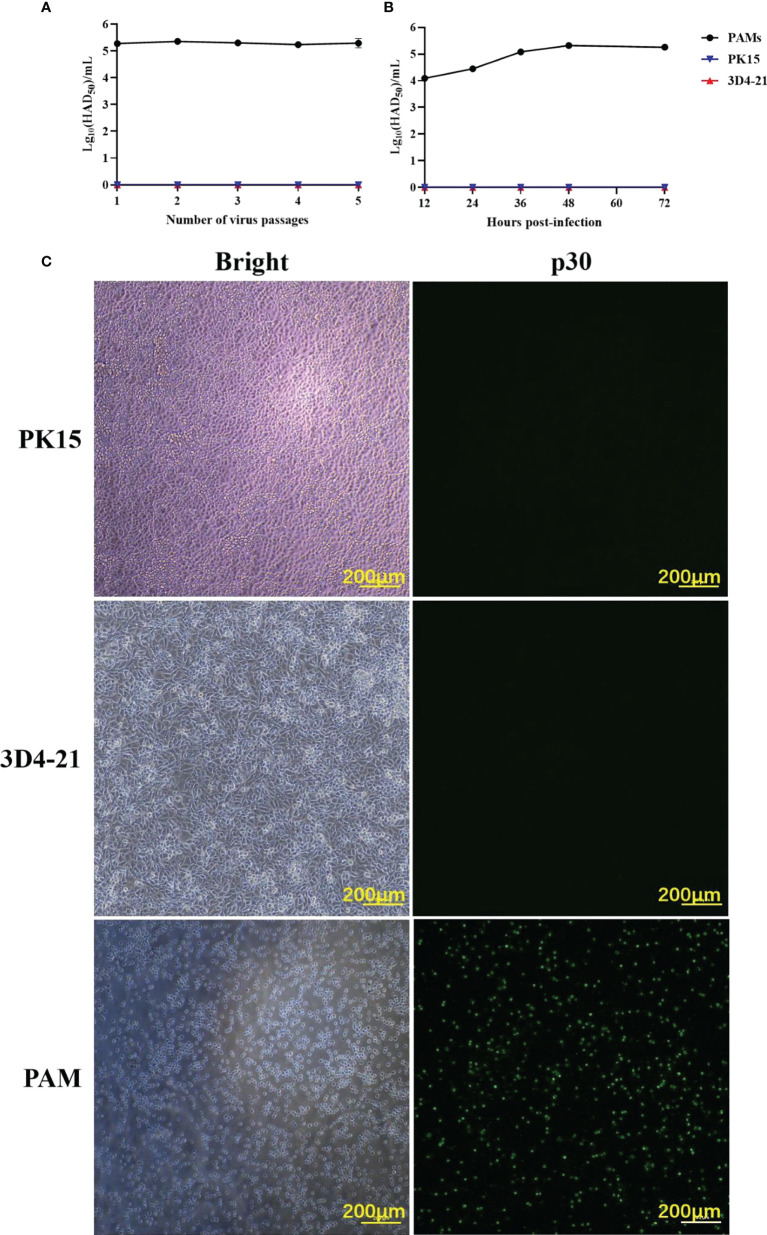
The ability of ASFV to infect PAMs, PK15, and 3D4-21 cells. **(A)** ASFV was serially passaged five times in PAM, PK15, and 3D4-21 cells. Monolayers of PAM, PK15, and 3D4/21 cells seeded in T25 cell culture flasks were infected with 1 MOI of ASFV and incubated at 37°C with 5% CO_2_ for five days. Cells and supernatants were collected and stored at -80°C. For the next four passages, cells were infected with 1 mL of the virus solution from the previous passage, and the virus was continuously passaged five times under the same conditions. The HAD_50_ of the each ASFV generation was detected. **(B)** Proliferation curves of ASFV in PAM, PK15, and 3D4-21 cells. After infecting PAM, PK15, and 3D4/21 cells with 1 MOI of ASFV for 12, 24, 36, 48, and 72 (h) The HAD_50_ of the each ASFV of ASFV in the collected samples were detected. **(C)** Immunofluorescence assay (IFA) results post-infection of PAM, PK15, and 3D4/21 cells with ASFV. PAM, PK15, and 3D4/21 cells were infected with 1 MOI of ASFV for 24 h, and viral p30 protein expression was detected using IFA.

To identify ASFV-susceptible cells, ASFV was serially passaged in PAM, PK15, and 3D4-21 cells for five passages, and it was found that ASFV maintained a consistently low virus titer only in PAMs (**Figure 1A**). After infecting PAM, PK15, and 3D4-21 cells with 1 MOI of ASFV for 12, 24, 36, 48, and 72 h, HAD_50_ results showed that ASFV proliferated efficiently in PAMs only (**Figure 1B**). Moreover, IFA results showed that p30 protein was only detected during ASFV infection of PAMs (**Figure 1C**). These results indicate that ASFV can infect PAMs, but not PK15 and 3D4-21 cells.

### Genome-wide transcriptional profiling of PAMs and PK15 and 3D4-21 cells

**Figure 2:**
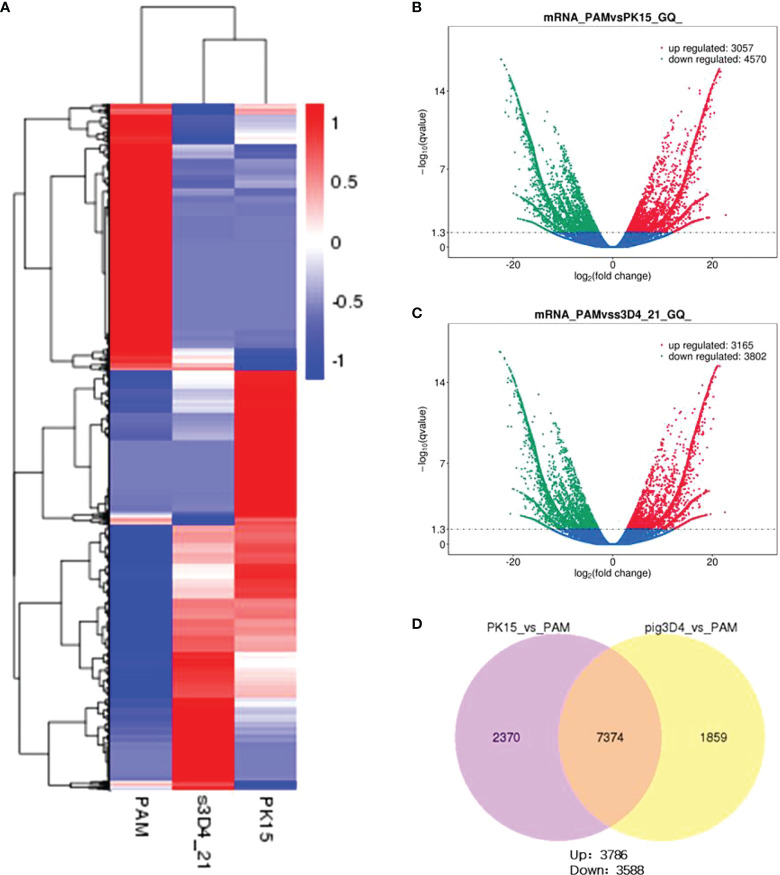
Transcriptomic analysis of PAM, PK15, and 3D4-21 cells. **(A)** Clustering heatmap analysis of PAM, PK15, and 3D4-21 cell transcriptional profiles. **(B)** Volcano plot analysis of genes with differential transcriptional profiles between PAM and PK15 cells. Genes downregulated (green) and upregulated (red) by PAMs were compared with the transcriptional profile of PK15 cells, genes with no significant difference are indicated in blue. **(C)** Volcano plot analysis of genes with differential transcriptional profiles between PAMs and 3D4-21 cells. Genes downregulated (green) and upregulated (red) by PAMs were compared with the transcriptional profile of 3D4-21 cells, genes with no significant difference are indicated in blue. **(D)** Venn diagram analysis of differential genes. Venn diagram analysis was performed on the differential genes of PK15 and PAM cells and 3D4-21 and PAM cells, showing the number of common differential genes and the number of upregulated and downregulated genes.

**Figure 3:**
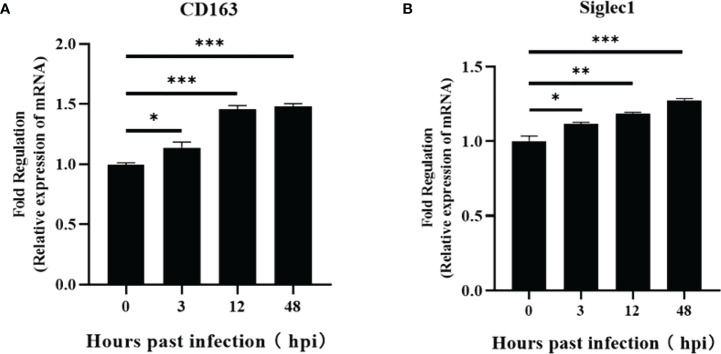
Regulation of CD163 and Siglec1 by ASFV-infected PAMs. Transcript levels of CD163 and Siglec1 were detected using RT-qPCR at 3, 12, 24, and 48 h post-infection of PAMs with ASFV **(A, B)**. Each datum represents results of three independent experiments (means ± SD). Significant differences compared with the control group are denoted by *p < 0.05, **p < 0.01, and ***p < 0.001.

To explore the influencing factors that limit the culture of ASFV in passaged cell lines, we performed transcriptomic sequencing of ASFV-susceptible PAMs and non-susceptible cell lines PK15 and 3D4-21 to obtain genome-wide transcriptional profiles. Bioinformatic clustering analysis of DEGs revealed that the transcribed genes of PK15 and 3D4-21 produced efficient clusters (**Figure 2A**). Compared with PAMs, the DEGs identified in PK15 cells included 4570 upregulated genes and 3057 downregulated genes (**Figure 2B**), while 3D4-21 cells displayed 3802 upregulated genes and 3165 downregulated genes (**Figure 2C**). Venn diagram analysis was performed between the DEGs of PK15 and PAM cells and 3D4-21 and PAM cells. Results showed that two comparison groups had 7374 common DEGs, including 3786 common upregulated DEGS and 3588 common downregulated DEGs (**Figure 2D**). GO functional enrichment analysis of 3588 commonly downregulated DEGs revealed that DEGs were significantly enriched in Endocytosis, Phagocytosis, Endosome, Positive regulation of phagocytosis, Regulation of endocytosis, Regulation of phagocytosis, Positive regulation of endocytosis, Receptor-mediated endocytosis, Endosome membrane, Phagocytosis, engulfment, Late endosome, Autophagosome, Early endosome, and other phagocytosis-related functions (**Table 1**). KEGG pathway enrichment analysis of 3588 commonly downregulated genes showed that DEGs were significantly enriched in Phagosome, Phagocytosis, and Endocytosis pathways (**Table 2**). Therefore, we compared the transcript levels of phagocytosis-related pattern recognition receptors CD163 and Siglec1 in PAM, PK15, and 3D4-21 cells, and observed higher transcript levels of CD163 and Siglec1 in PAMs. However, these two genes were not present in PK15 and 3D4-21 cells (**Table 3**). Using RT-qPCR at 3, 12, 24, and 48 h post-ASFV infection, we observed significantly increased transcription levels of CD163 and Siglec1 in PAMs, as ASFV infection time increased (**Figure 3**). Our results indicate that ASFV infection may significantly correlate with the expression levels of endocytosis-related pattern recognition receptors CD163 and Siglec1. Therefore, the functions of CD163 and Siglec1 in ASFV-infected cell lines were investigated.

**Table 1:** Gene Ontology (GO) functional enrichment analysis of common differential genes in PK15 and 3D4-21 cell lines and porcine alveolar macrophages (PAMs).

GO	ID	Count	p-Value
Endocytosis	0006897	163	2.71E-20
Phagocytosis	0006909	65	1.65E-16
Endosome	0005768	172	2.02E-14
Positive regulation of phagocytosis	0050766	24	3.56E-09
Regulation of endocytosis	0030100	63	1.77E-08
Regulation of phagocytosis	0050764	29	2.49E-08
Positive regulation of endocytosis	0045807	43	5.18E-08
Receptor-mediated endocytosis	0006898	68	5.99E-08
Endosome membrane	0010008	57	3.44E-06
Phagocytosis, engulfment	0006911	17	7.51E-06
Late endosome	0005770	45	9.06E-06
Autophagosome	0005776	30	1.10E-05
Early endosome	0005769	56	0.00016796

**Table 2:** Kyoto Encyclopedia of Genes and Genomes (KEGG) pathway enrichment analysis of common differential genes in PK15 and 3D4-21 cell lines and porcine alveolar macrophages (PAMs).

KEGG PATHWAY	ID	Count	p-Value
Phagosome	ssc04145	82	1.80E-06
Phagocytosis	ssc04666	53	4.49E-06
Endocytosis	ssc04144	74	0.053331676

**Table 3:** CD163 and Siglec1 levels in porcine alveolar macrophages (PAMs) and PK15 and 3D4-21 cells, respectively, including the FPKM, fold change and p-value of the content in different cells.

	CD163	Siglec1
PAM_FPKM	270.0117887	39.070276
PK15_FPKM	0.011321	0.005022
3D4-21_FPKM	0	0.016200667
PAM vs PK15 log2 Fold Change	19.17263322	13.3386979
PAM vs 3D4-21 log2 Fold Change	22.03440971	11.3778428
PAM vs PK15 p-value	0	4.56E-133
PAM vs 3D4-21 p-value	0	5.67E-146

### Effects of CD163 and Siglec1 on ASFV infection

**Figure 4:**
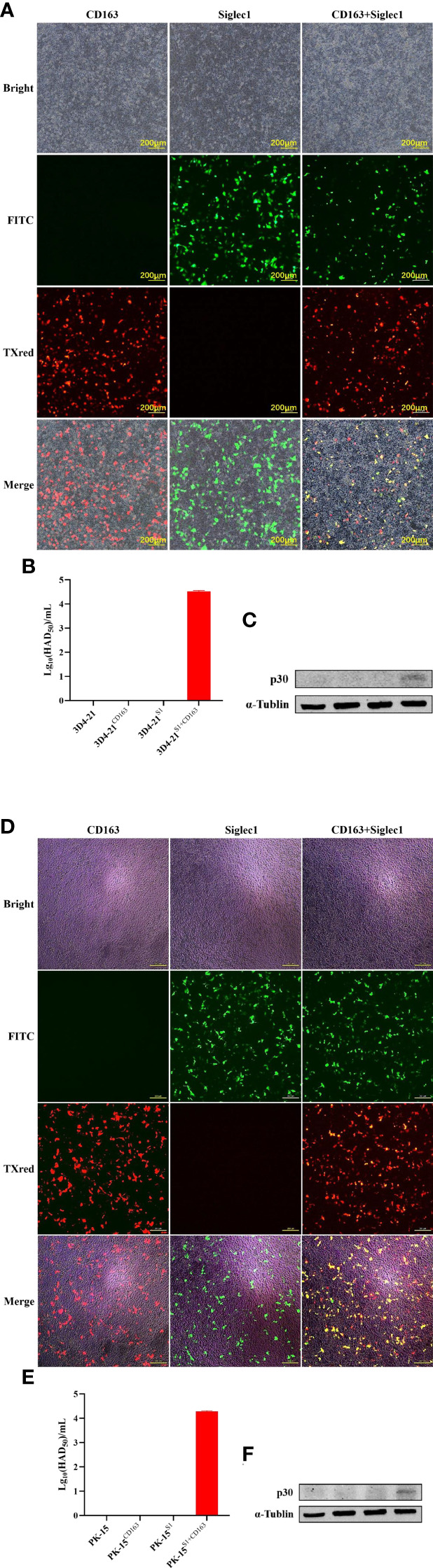
Effects of CD163 and Siglec1 on ASFV-infected cell lines *in vitro*. CD163 and Siglec1 were transfected into PK15 and 3D4-21 cells separately or together, and the expression of mCherry-tagged red fluorescent protein CD163 and eGFP-tagged green fluorescent protein Siglec1 was observed using a fluorescence microscope **(A–D)**. After CD163 and Siglec1 were transfected into PK15 and 3D4-21 cells, the cells were infected with ASFV, the HAD_50_ of ASFV was detected **(B–E)**, and the expression level of ASFV p30 protein was detected using western blot **(C–F)** . Each datum represents results of three independent experiments (means ± SD).

Plasmids expressing CD163 and Siglec1 were respectively or co-transfected into PK15 and 3D4-21 cell lines (**Figures 4A, D**), and the infectivity of ASFV was detected 48 h after inoculating cells with 1 MOI of ASFV. HAD_50_ results showed that increased virus titer was observed in ASFV-infected PK15 and 3D4-21 cells simultaneously overexpressing CD163 and Siglec1, compared to PK15 and 3D4-21 cells overexpressing CD163 and Siglec1, respectively. HAD50 results showed that ASFV can infect PK15 and 3D4-21 cells overexpressing both CD163 and Siglec1 (**Figures 4B, E**). Western blot results showed that viral p30 protein could be detected after ASFV infection in PK15 and 3D4-21 cells overexpressing both CD163 and Siglec1 (**Figures 4C, F**). These findings indicate that CD163 and Siglec1 have a synergistic effect on the ability of ASFV to infect cells *in vitro*, and are common coreceptors for ASFV to infect host cells.

### Infectivity of ASFV in PK15^S1-CD163^ cells

**Figure 5:**
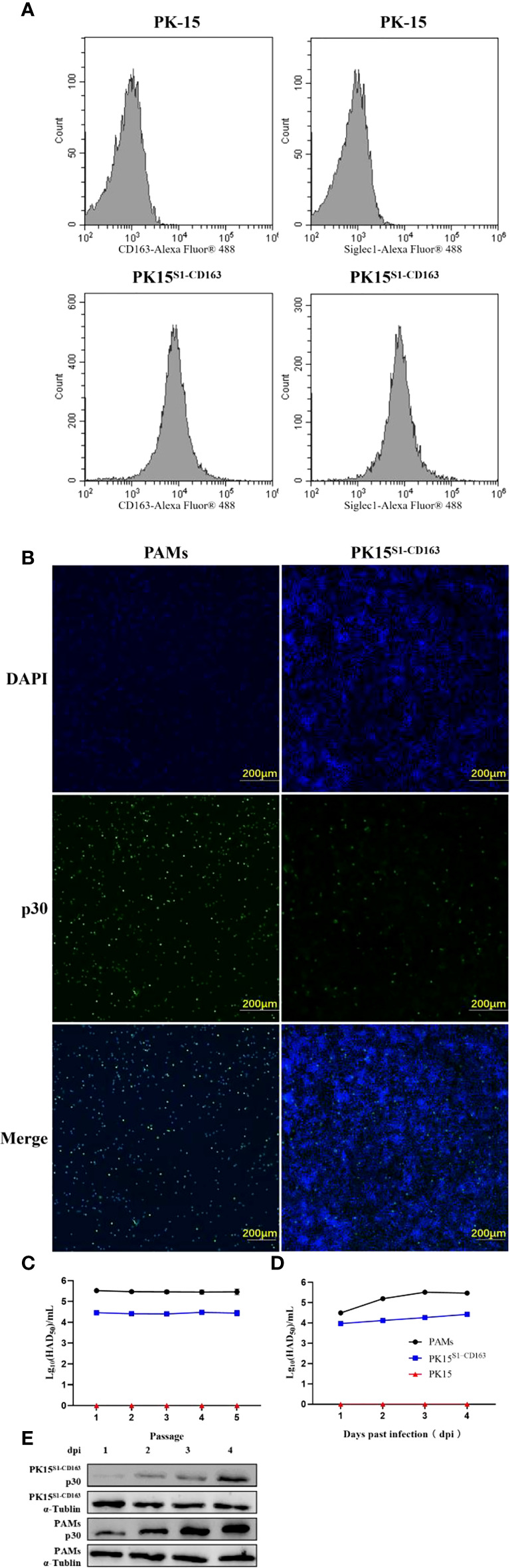
ASFV is infectious to PK15^S1-CD163^ cells stably expressing CD163 and Siglec1. **(A)** The expressions of CD163 and Siglec1 in PK15 and PK15^S1-CD163^ cell lines were detected by flow cytometry. **(B)** ASFV (1 MOI) infected PAM, PK15^S1-CD163^ and PK15 cells, and the expression of ASFV p30 protein was detected using IFA. **(C)** ASFV was blindly passaged in PAM, PK15^S1-CD163^ and PK15 cells five times, and the HAD_50_ of ASFV in the five passages were detected. **(D)** The HAD_50_ of ASFV in the cells was detected after PAM and PK15^S1-CD163^ cells were infected with ASFV for one, two, three, and four days. **(E)** Western blot was used to detect the protein expression of ASFV p30 after PAM and PK15^S1-CD163^ cells were infected with ASFV for one, two, three, and four days.

The expressions of CD163 and Siglec1 in PK15 and PK15^S1-CD163^ cell lines were detected by flow cytometry (**Figure 5A**). The results showed that CD163 and Siglec1 proteins were stably expressed in the PK15^S1-CD163^ cell line. ASFV (1 MOI) was used to infect PAMs, PK15^S1-CD163^ and PK15 cells stably expressing CD163 and Siglec1, respectively. After 24 h, ASFV p30 protein was detected using IFA. Compared with PAMs, PK15^S1-CD163^ and PK15 cells displayed a relatively low level of p30 protein expression after ASFV infection (**Figure 5B**). PAMs were serially passaged for 5 passages using 1 MOI of ASFV, and HAD_50_ assays were performed on infected cells at each passage. The infectivity in PK15^S1-CD163^ cells was lower than that observed in PAMs (**Figure 5C**). HAD_50_ results one two, three, and four days post-ASFV infection showed that ASFV displayed replication ability in both cell lines, but this replication was more robust in PAMs than that in PK15^S1-CD163^ cells (**Figure 5D**). Western blot experiments were performed on PK15^S1-CD163^ and PAM cells one, two, three, and four days post-infection with 1 MOI ASFV, and showed that p30 protein expression increased as the infection time increased. Furthermore, p30 protein expression in ASFV-infected PAMs was significantly higher than that in PK15^S1-CD163^ cells (**Figure 5E**). The above results indicate that ASFV can infect the PK15^S1-CD163^ cells stably expressing CD163 and Siglec1 and can replicate and stably passage in this cell line.

### Infectivity of ASFV in PAMs with silenced CD163 and Siglec1

**Figure 6:**
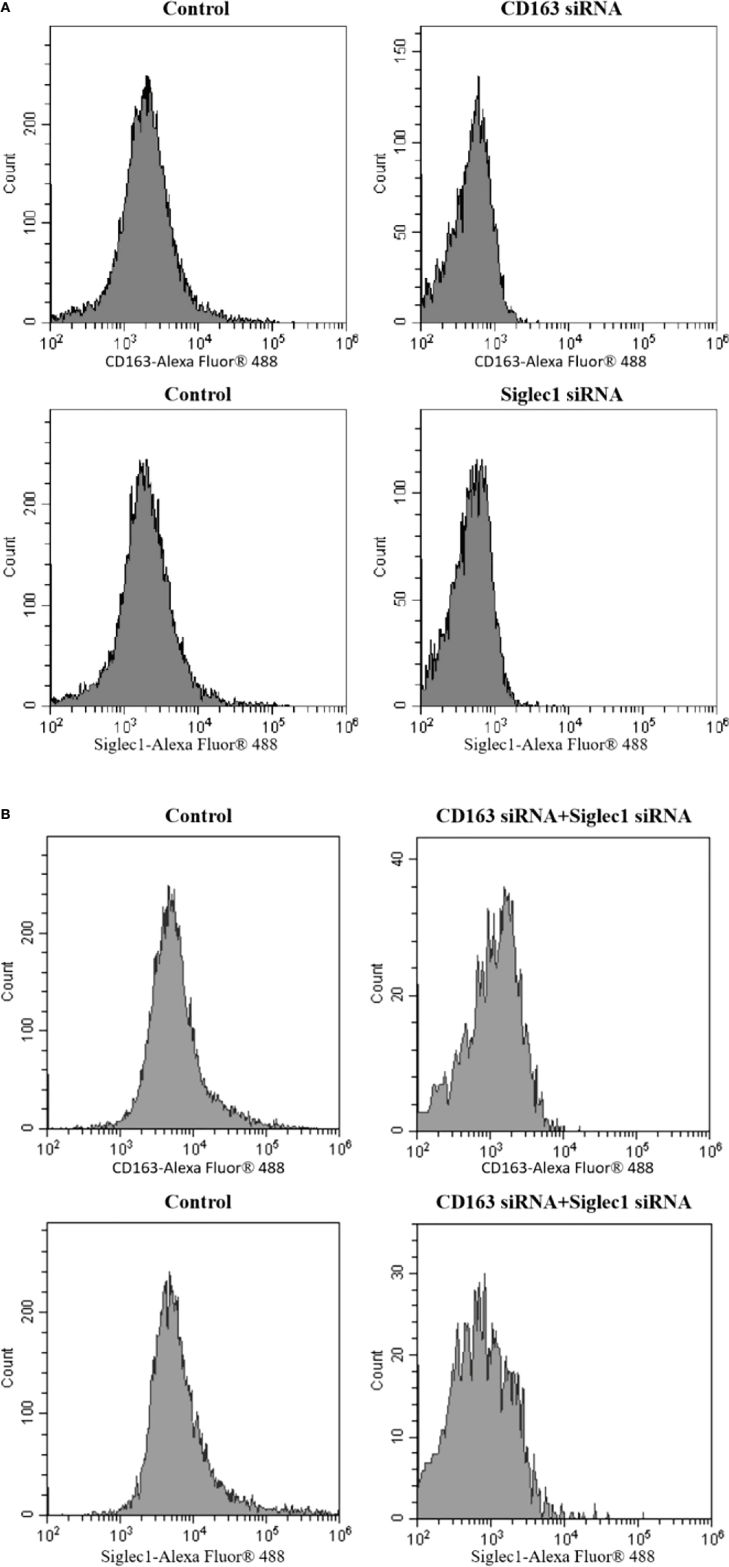
The CD163 and Siglec1 levels in PAMs were silenced by siRNA, which was detected using flow cytometry. **(A)** The PAMs were silenced by CD163 siRNA and Siglec1 siRNA, respectively, and the expression of CD163 and Siglec1 was detected. **(B)** Simultaneously, CD163 siRNA and Siglec1 siRNA were used to silence PAMs, and the expressions of CD163 and Siglec1 were detected.

**Figure 7:**
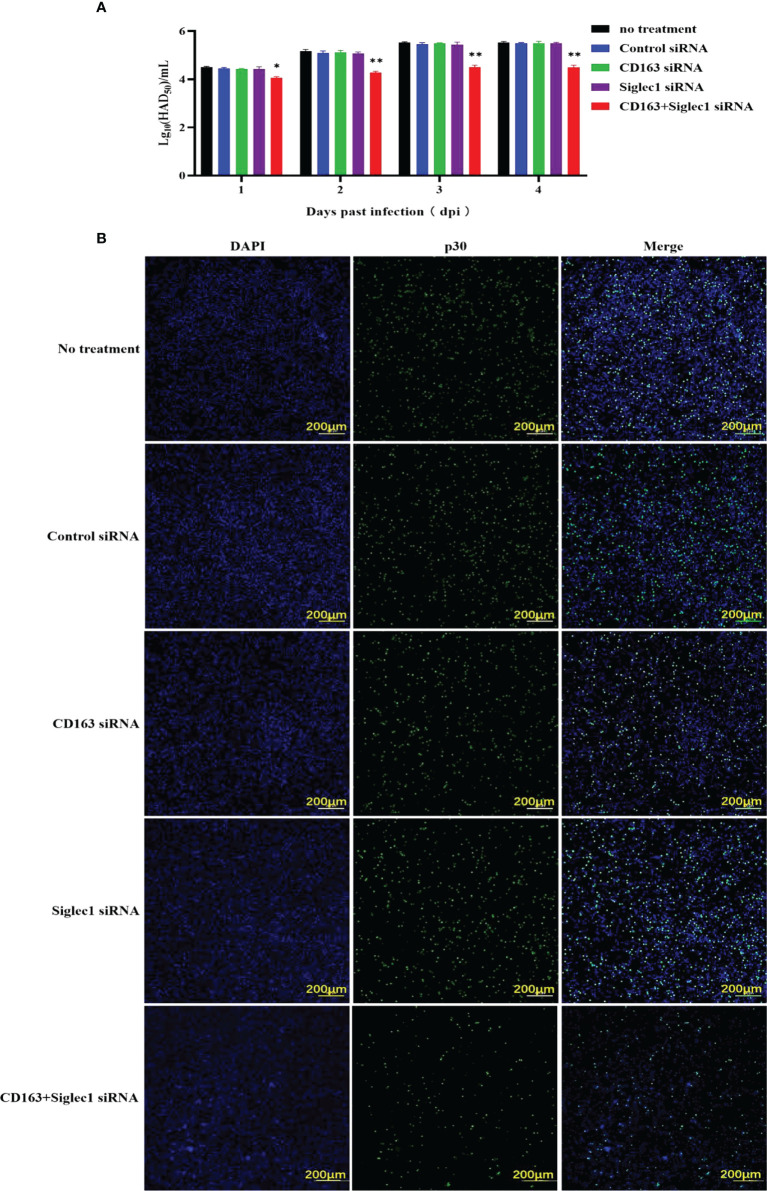
Infectious capacity of ASFV in PAMs with silenced CD163 and Siglec1. **(A)** CD163 and Siglec1 were silenced in PAMs using siRNA, and the HAD_50_ of ASFV-infected PAMs was detected. **(B)** CD163 and Siglec1 were silenced in PAMs and p30 protein expression levels in ASFV-infected PAMs were detected using IFA. Each datum represents results of three independent experiments (means ± SD). Significant differences compared with the control group are denoted by *p < 0.05, and **p < 0.01.

siRNA was used to silence CD163 and Siglec1 in PAMs cells, which was confirmed using flow cytometry (**Figures 6A, B**). Next, CD163 and Siglec1 silenced PAMs were infected with ASFV and subjected to HAD_50_. ASFV infectivity was significantly reduced only when both CD163 and Siglec1 were silenced (**Figure 7A**). Using IFA, we observed that p30 protein expression was significantly decreased only when CD163 and Siglec1 were silenced simultaneously (**Figure 7B**). These results demonstrate that CD163 and Siglec1 act as key membrane protein receptors during ASFV infection.

### ASFV inhibits type I IFN in PAMs and PK15^S1-CD163^ cells

**Figure 8:**
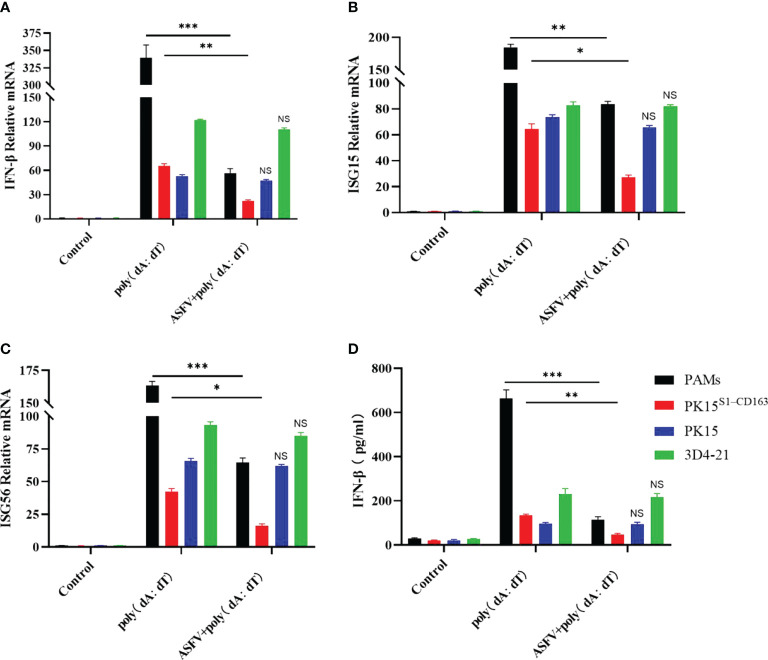
The regulatory effect of ASFV on host type I IFN. PK15, 3D4-21, PAM, and PK15^S1-CD163^ cells were infected with 1 MOI of ASFV, respectively, and levels of IFN-β **(A)**, ISG15 **(B)**, and ISG56 **(C)** genes were detected in the cells 24 h later using qPCR. **(D)** The supernatants were collected, and the amounts of secreted IFN-β protein were measured using an ELISA kit. Each datum represents results of three independent experiments (means ± SD). Significant differences compared with the control group are denoted by *p < 0.05, **p < 0.01, and ***p < 0.001. No Significance (NS) indicates that there is No statistical difference between data.

PK15, 3D4-21, PAM and PK15^S1-CD163^ cells were inoculated with ASFV (1 MOI) for 24 h to detect the expression of type I IFN in cells. We observed that ASFV infection inhibited the transcription level of IFN-β in PAMs and PK15^S1-CD163^ cells (**Figure 8A**), while the transcriptional levels of ISG15 and ISG56, which regulate IFN-mediated antiviral effects, were also inhibited. Moreover, transcriptional levels of IFN-β, ISG15, and ISG56 were not inhibited in PK15 and 3D4-21 cells (**Figures 8B, C**). The supernatants were collected, and the amounts of secreted IFN-β protein were measured using an ELISA kit. ASFV also inhibited IFN-β protein secretion only in PAMs and PK15^S1-CD163^ cells (**Figure 8D**). The above results indicate that ASFV significantly inhibits the antiviral effect of the host IFN pathway after infecting PAMs and PK15^S1-CD163^ cells.

## Discussion

Since the susceptible cells of the wild ASFV strain are primary porcine mononuclear macrophages, which are generally not suitable for propagation in *in vitro* passaged cell lines, this limits the study of ASFV virus-host interactions, pathogenicity research, and vaccine development and production. In recent years, researchers have established and transformed porcine mononuclear cell lines for detection, growth, and titer studies of isolated strains, but stable cell lines infected with ASFV *in vitro* have not been established. In this study, by comparing the genome-wide transcriptional profiles of ASFV-susceptible PAMs and non-susceptible cell lines, PK15 and 3D4-21, we observed that endocytosis of CD163 and Siglec1 by host cell surface PRRs plays a key role in ASFV infection *in vitro*. CD163 is a cell surface glycoprotein receptor expressed in peripheral blood monocytes and macrophages in most tissues (28), a target for pathogen entry into cells and a receptor that mediates endocytosis (29). Studies have shown that CD163 can activate inflammatory pathways and stimulate the host to produce pro-inflammatory factors (16). In the process of porcine reproductive and respiratory syndrome virus (PRRSV) invasion of host target cells, CD163 plays an important role, and was identified as a receptor for PRRSV (30). CD163 is also considered as an ASFV receptor. When porcine monocytes (PBMCs) differentiate into mature macrophages, CD163 expression increases, and ASFV infection also increases (20, 21). CD163 gene knockout pigs display resistance to infection by the Georgia07 strain. In addition, studies have shown that anti-CD163-specific antibodies reduce ASFV infection after incubation with macrophages (22). However, the role of CD163 in ASFV infection is also controversial, and studies have shown that stable expression of CD163 in less susceptible cell lines does not increase the infectivity of ASFV (23). Here, we overexpressed CD163 in 3D4-21 and PK15 cell lines, determined ASFV infection, and found that ASFV was not infective to CD163-overexpressing 3D4-21 and PK15 cells. We used CD163 siRNA in PAMs, and found that silencing CD163 expression did not produce a significant effect on ASFV infection. Therefore, it is speculated that CD163 is not the only receptor for ASFV-infected hosts. Sialadhesin (Siglec1) is an adhesion molecule of the immunoglobulin superfamily expressed on macrophages. Siglec1 regulates the secretion of cytokines and promotes pro-inflammatory responses. Siglec1, as a marker of macrophage activation, has also been further studied for its role in inflammatory response and immune regulation. Siglec1 promotes viral infection and phagocytosis by mediating the combination of pathogens and macrophages. Siglec1 also inhibits the overexpression of IFN during the immune response, thereby inhibiting the host’s innate immunity and adaptive immunity. During SARS-CoV-2 infection, Siglec1 reportedly acts as an adsorption receptor to enhance the ability of ACE2 to attach to the infected host (31). Macrophages can rely on Siglec1-mediated phagocytosis for Neisseria meningitidis infection (24–26). Viruses, such as HSV, increase Siglec1 expression in macrophages through the IFN-JAK-STAT1 pathway, which negatively regulates the overexpression of IFN, thereby inhibiting the innate immune response and enhancing immune escape by the virus (32). PRRSV can bind to Siglec1 *via* sialylated viral glycoproteins (33), and Siglec1 can promote PRRSV infection by inhibiting the production of IFN-1 (34). Therefore, macrophages expressing Siglec1 can exert phagocytosis to facilitate viral infection.

We found that ASFV was not infective to PK15 and 3D4-21 cells overexpressing Siglec1, and silencing Siglec1 in PAMs had no significant effect on ASFV infection. In contrast, ASFV showed obvious infectivity to PK15 and 3D4-21 cells overexpressing both CD163 and Siglec1, or the PK15 cell line stably expressing CD163 and Siglec1. Silencing CD163 and Siglec1 in PAMs significantly inhibited ASFV infection. These results are consistent with reports that Siglec1 and CD163 are key receptors involved in viral attachment, internalization, and uncoating (27, 35–37). This experiment also found that ASFV infection of PAMs and PK15^S1-CD163^ cells significantly inhibits the production of type I IFN. This agrees with previously reported results indicating that ASFV inhibits type I IFN in infected cells. According to reports that Sialec-1 inhibits overexpression of IFN during immune regulation, thereby suppressing the innate and adaptive immune responses and promoting viral infection (38, 39). Therefore, we speculate that Siglec1 has an important regulatory role in promoting ASFV infection. The above results revealed the synergistic effect of CD163 and Siglec1 in ASFV infection and improve our understanding of the pathogenic mechanism underlying ASFV infection. However, the ASFV genome is long and complex in structure. Most of the encoded proteins have unknown functions, and their related host receptors are also rarely studied, how CD163 and Siglec1 act as ASFV receptors to facilitate viral invasion is currently unknown. Therefore, the pathway through which CD163 and Siglec1 induce ASFV invasion of host cells requires further study. Next, our laboratory will further study the roles of CD163 and Siglec1 in ASFV invasion to establish a more complete *in vitro* cell line that can be infected with ASFV, and provides theoretical support for the mechanism underlying ASFV infection and the development of vaccines.

## Data availability statement

The data presented in the study are deposited in the NCBI SRA repository, accession number PRJNA883625.

## Author contributions

QG analyzed the data and drafted the manuscript. YY, YL, JZ, RW, LG and HW carried out most of the experiments. YF, TG, DW, and XZ participated in the study design. GZ and ZZ conceived the study. All authors have read and agreed to the published version of the manuscript.

## Funding

This work was supported by the National Key Research and Development Program of China (grant number 2021YFD1800100), Start-up Research Project of Maoming Laboratory (2021TDQD002), and China Agriculture Research System of MOF and MARA (cars-35).

## Acknowledgments

Thanks to Prof. Hans J. Nauwynck, Department of Virology, Immunology and Parasitology, Faculty of Veterinary Medicine, Ghent University, Belgium, for the gift of the PK15^S1-CD163^ cell line.

## Conflict of interest

The authors declare that the research was conducted in the absence of any commercial or financial relationships that could be construed as a potential conflict of interest.

## Publisher’s note

All claims expressed in this article are solely those of the authors and do not necessarily represent those of their affiliated organizations, or those of the publisher, the editors and the reviewers. Any product that may be evaluated in this article, or claim that may be made by its manufacturer, is not guaranteed or endorsed by the publisher.

## References

[1] Revilla Y Perez-Nunez D Richt JA. African Swine fever virus biology and vaccine approaches. Adv Virus Res (2018) 100:41–74. doi: 10.1016/bs.aivir.2017.10.002 29551143

[2] Dixon LK Chapman DA Netherton CL Upton C. African Swine fever virus replication and genomics. Virus Res (2013) 173:13–4. doi: 10.1016/j.virusres.2012.10.020 23142553

[3] Borca MV Rai A Ramirez-Medina E Silva E Velazquez-Salinas L Vuono E. A cell culture-adapted vaccine virus against the current African swine fever virus pandemic strain. J Virol (2021) 95:e0012321. doi: 10.1128/JVI.00123-21 33952643 PMC8315737

[4] Wang T Wang L Han Y Pan L Yang J Sun M. Adaptation of African swine fever virus to HEK293T cells. Transbound Emerg Dis (2021) 68:2853–66. doi: 10.1111/tbed.14242 34314096

[5] Alejo A Matamoros T Guerra M Andrés G. A proteomic atlas of the African swine fever virus particle. J Virol (2018) 92:e01293–18. doi: 10.1128/JVI.01293-18 PMC6232493 30185597

[6] Galindo I Alonso C. African Swine fever virus: a review. Viruses (2017) 9:103. doi: 10.3390/v9050103 PMC5454416 28489063

[7] Dixon LK Sun H Roberts H. African Swine fever. Antiviral Res (2019) 165:34–41. doi: 10.1016/j.antiviral.2019.02.018 30836106

[8] Gómez-Villamandos JC Bautista MJ Sánchez-Cordón PJ Carrasco L. Pathologyof African swine fever: The role of monocyte-macrophage. Virus Res (2013) 173:140–9. doi: 10.1016/j.virusres.2013.01.017 23376310

[9] Wöhnke E Fuchs W Hartmann L Bloh U Blome S Mettenleiter TC. Comparison of the proteomes of porcine macrophages and a stable porcine cell line after infection with African swine fever virus. Viruses (2021) 13:2198. doi: 10.3390/v13112198 34835004 PMC8620826

[10] Balysheva VI Prudnikova EY Galnbek TV Balyshev VM. Continuous cell subline A4C2/9K and its application to the african swine fever virus study. Voprosy Virusol (2015) 60:43–7. 26182658

[11] Masujin K Kitamura T Kameyama KI Okadera K Nishi T Takenouchi T. An immortalized porcine macrophage cell line competent for the isolation of African swine fever virus. Sci Rep (2021) 26:11. 4759. doi: 10.1038/s41598-021-84237-2 PMC7910288 33637799

[12] Portugal R Goatley LC Husmann R Zuckermann FA Dixon LK. A porcine macrophage cell lin that supports high levels of replication of OURT88/3, an attenuated strain of African swine fever virus. Emerg Microbes infect (2020) 9:1245–53. doi: 10.1080/22221751.2020.1772675 PMC7448849 32515659

[13] Ayushi R Sarah P Elizabeth RM Elizabeth AV Ediane S Lauro VS. Detection and quantification of African swine fever virus in MA-104 cells. Bio-protocol (2021) 20(11):e3955. doi: 10.21769/BioProtoc.3955 PMC8032485 33855107

[14] Andrés G. African Swine fever virus gets undressed: New insights on the entry pathway. J Virol (2017) 91:e01906–16. doi: 10.1128/JVI.01906-16 PMC5286891 27974557

[15] Adolfo GS. Influenza virus receptor specificity: disease and transmission. Am J Pathol (2010) 176:1584–5. doi: 10.2353/ajpath.2010.100066 PMC2843447 20203283

[16] Law SK Micklem KJ Shaw JM Zhang XP Dong Y Willis AC. A new macrophage differentiation antigen which is a member of the scavenger receptor superfamily. Eur J Immunol (1993) 23:2320–5. doi: 10.1002/eji.1830230940 8370408

[17] Ritter M Buechler C Langmann T Schmitz G. Genomic organization and chromosomal localization of the human CD163 (M130) gene: A member of the scavenger receptor cysteine-rich superfamily. Biochem Biophys Res Commun (1999) 260:466–74. doi: 10.1006/bbrc.1999.0866 10403791

[18] Calvert JG Slade DE Shields SL Jolie R Mannan RM Ankenbauer RG. CD163 expression confers susceptibility to porcine reproductive and respiratory syndrome viruses. J Virol (2007) 81:7371–9. doi: 10.1128/JVI.00513-07 PMC1933360 17494075

[19] Backe E Schwarting R Gerdes J Ernst M Stein H. Ber-MAC3: New monoclonal antibody that defines human monocyte/macrophage differentiation antigen. J Clin Pathol (1991) 44:936–45. doi: 10.1136/jcp.44.11.936 PMC496636 1721628

[20] Chamorro S Revilla C Alvarez B López-Fuertes L Ezquerra A Domínguez J. Phenotypic characterization of monocyte subpopulations in the pig. Immunobiology (2000) 202:90–3. doi: 10.1016/S0171-2985(00)80055-8 10879692

[21] McCullough KC Basta S Knötig S Gerber H Schaffner R Kim KB. A summerfield intermediate stages in monocyte macrophage differentiation modulate phenotype and susceptibility to virus infection. Immunology (1999) 98(2):203–12. doi: 10.1046/j.1365-2567.1999.00867.x PMC2326918 10540219

[22] Lithgow P Takamatsu H Werling D Dixon L Chapman D. Correlation of cell surface marker expression with African swine fever virus infection. Vet Microbiol (2014) 168:413–9. doi: 10.1016/j.vetmic.2013.12.001 PMC3969584 24398227

[23] Popescu L Natasha NG Whitworth KM Murgia MV Nietfeld JC Mileham A. Genetically edited pigs lacking CD163 show no resistance following infection with the African swine fever virus isolateGeorgia2007/1. Virology (2017) 501:102–6. doi: 10.1016/j.virol.2016.11.012 27898335

[24] Hartnell A Steel J Turley H Jones M Jackson DG Crocker PR. Characterization of human sialoadhesin,a sialic acid binding receptor expressed by resident and inflammatory macrophage populations. Blood (2001) 97:288–96. doi: 10.1182/blood.V97.1.288 11133773

[25] Munday J Floyd H Crocker PR. Sialic acid bindingreceptors (siglecs) expressed by macrophages. J leukocyte Biol (1999) 66:705–11. doi: 10.1002/jlb.66.5.705 10577497

[26] Crocker PR Paulson JC Varki A. Siglecs and their roles in the immune system. Nat Rev Immunol (2007) 7:255–66. doi: 10.1038/nri2056 17380156

[27] Vanderheijden N Delputte PL Favoreel HW Vandekerckhove J Damme JV Woensel PAV. Involvement of sialoadhesin in entry of porcine reproductive and respiratory syndrome virus into porcine alveolar macrophages. J Virlogy (2003) 77:8207–15. doi: 10.1128/JVI.77.15.8207-8215.2003 PMC165228 12857889

[28] Chen JY Wang HT Bai JH Liu WJ Liu XJ Yu DW. Generation of pigs resistant to highly pathogenic-porcine reproductive and respiratory syndrome virus through gene editing of CD163. Int J Biol Sci (2019) 15:481–92. doi: 10.7150/ijbs.25862 PMC6367541 30745836

[29] Melamed-Frank M Lache O Enav BI Szafranek T Levy NS Ricklis RM. Structure-function analysis of the antioxidant properties of haptoglobin. Blood (2001) 15, 98:3693–8. doi: 10.1182/blood.V98.13.3693 11739174

[30] Welch SKW Calvert JG. A brief review of CD163 and its role in PRRSV infection. Virus Res (2010) 154:98–103. doi: 10.1016/j.virusres.2010.07.018 20655964

[31] Lempp FA Soriaga LB Montiel-Ruiz M Benigni F Noack J Park YJ. Lectins enhance SARS-CoV-2 infection and influence neutralizing antibodies. Nature (2021) 598:342–7. doi: 10.1038/s41586-021-03925-1 34464958

[32] Langevin C Aa LM Houel A Torhy C Briolat V Lunazzi A. Zebrafish ISG15 exerts a strong antiviral activity against RNA and DNA viruses and regulates the interferon response. J Virol (2013) 87:10025–36. doi: 10.1128/JVI.01294-12 PMC3753986 23824820

[33] Xie JX Christiaens I Yang B Trus I Devriendt B Cui TT. Preferential use of siglec-1 or siglec-10 by type 1 and type 2 PRRSV strains to infect PK15 S1-CD163 and PK15 S10-CD163 cells. Vet Res (2018) 49:67. doi: 10.1186/s13567-018-0569-z 30021620 PMC6052533

[34] Zhang LJ Xu RQ Wei FL Li W Li XT Zhang GP. Activation of sialoadhesin down-regulates the levels of innate antiviral cytokines in porcine alveolar macrophages *in vitro* . Virus Res (2020) 275:197792. doi: 10.1016/j.virusres.2019.197792 31669458

[35] Duan X Nauwynck HJ Favoreel HW Pensaert MB. Identification of a putative receptor for porcine reproductive and respiratory syndrome virus on porcine alveolar macrophages. J Virlogy (1998) 72:4520–3. doi: 10.1128/JVI.72.5.4520-4523.1998 PMC109698 9557752

[36] Delputte PL Meerts P Costers S Nauwynck HJ. Effect of virus-specific antibodies on attachment, internalization and infection of porcine reproductive and respiratory virus in primary macrophages. Vet Immunol immunopathol (2004) 102:179–88. doi: 10.1016/j.vetimm.2004.09.007 15507304

[37] Gorp HV Breedam WV Delputte PL Nauwynck HJ. Sialoadhesin and CD163 join forces during entry of the porcine reproductive and respiratory síndrome virus. J Gen Virol (2008) 89:2943–53. doi: 10.1099/vir.0.2008/005009-0 19008379

[38] Zheng QL Hou J Zhou Y Yang YY Xie B Cao XT. Siglec1 suppresses antiviral innate immune response by inducing TBK1 degradation *via* the ubiquitin ligase TRIM27. Cell Res (2015) 25:1121–36. doi: 10.1038/cr.2015.108 PMC4650625 26358190

[39] Akiyama H Ramirez NP Gibson G Kline C Watkins S Ambrose Z. Interferon-inducible CD169/Siglec1 attenuates anti-HIV-1 effects of alpha interferon. J Virol (2017) 91:e00972–17. doi: 10.1128/JVI.00972-17 PMC5640826 28794041

