# Supplementary material for: Adaptation of African swine fever virus to porcine kidney cells stably expressing CD163 and Siglec1
Source: Front Immunol. 2022 Oct 27;13:1015224. doi: 10.3389/fimmu.2022.1015224 (PMC9647134; doi:10.3389/fimmu.2022.1015224)
Supplement: Supplementary file 1 [file Table_1.docx]

**Supplementary file 1. Primers for genotyping**

| Primer sets | Sequences (5’–3’) |
| --- | --- |
| B646L | F: ATAGAGATACAGCTCTTCCAG  R: GTATGTAAGAGCTGCAGAAC |
| B646L-Probe | FAM-TATCGATAAGATTGAT-MGB |
| CD163 | F: AAGCCCACTGTAGGCAGAA  R: CCCCAGGAGGGAAACCAC |
| Sn F | F: CAACAAAGGAGCAGCAAT  R: TGGAGCATCTCGTGGATA |
| IFN-β | F: CAACAAAGGAGCAGCAAT  R: TGGAGCATCTCGTGGATA |
| ISG15 | F: GGTGCAAAGCTTCAGAGACC  R: GTCAGCCAGACCTCATAGGC |
| ISG56 | F: CCCACTTCTGTCTTACTGC  R: TACATTCTTGCCAGGTCTA |
| GAPDH | F:CCTTCCGTGTCCCTACTGCCAAC  R:GACGCCTGCTTCACCACCTTCT |
